# A pursuit of lineage-specific and niche-specific proteome features in the world of archaea

**DOI:** 10.1186/1471-2164-13-236

**Published:** 2012-06-12

**Authors:** Anindya Roy Chowdhury, Chitra Dutta

**Affiliations:** 1 Structural Biology & Bioinformatics Division, CSIR Indian Institute of Chemical Biology, 4, Raja S. C. Mullick Road, Kolkata, 700032, India

**Keywords:** Amino acid usage, Isoelectric point, COG distribution, Methanogen, Sulphur metaboliser, Korarachaeota, Oxygen requirement

## Abstract

**Background:**

Archaea evoke interest among researchers for two enigmatic characteristics –a combination of bacterial and eukaryotic components in their molecular architectures and an enormous diversity in their life-style and metabolic capabilities. Despite considerable research efforts, lineage- specific/niche-specific molecular features of the whole archaeal world are yet to be fully unveiled. The study offers the first large-scale *in silico* proteome analysis of all archaeal species of known genome sequences with a special emphasis on methanogenic and sulphur-metabolising archaea.

**Results:**

Overall amino acid usage in archaea is dominated by GC-bias. But the environmental factors like oxygen requirement or thermal adaptation seem to play important roles in selection of residues with no GC-bias at the codon level. All methanogens, irrespective of their thermal/salt adaptation, show higher usage of Cys and have relatively acidic proteomes, while the proteomes of sulphur-metabolisers have higher aromaticity and more positive charges. Despite of exhibiting thermophilic life-style, korarchaeota possesses an acidic proteome. Among the distinct trends prevailing in COGs (Cluster of Orthologous Groups of proteins) distribution profiles, crenarchaeal organisms display higher intra-order variations in COGs repertoire, especially in the metabolic ones, as compared to euryarchaea. All methanogens are characterised by a presence of 22 exclusive COGs.

**Conclusions:**

Divergences in amino acid usage, aromaticity/charge profiles and COG repertoire among methanogens and sulphur-metabolisers, aerobic and anaerobic archaea or korarchaeota and nanoarchaeota, as elucidated in the present study, point towards the presence of distinct molecular strategies for niche specialization in the archaeal world.

## Background

Over the past few decades, the process of establishing archaea as the third domain of life has been a stunning event in the world of life science. The world became familiar with this kingdom in 1977, when Woese & Fox [1] first proposed archaebacteria (subsequently renamed archaea) as a major domain - distinct from bacteria and eukaryotes but on equal footing with them. Prior to this three domain classification of life, which has been described by Makarova & Koonin [2] as “arguably one of the most important scientific discoveries of the twentieth century”; many of the ‘would-be’ archaea, used to be grouped under the bacterial lineage [2-4]. Phylogenetic analyses of rRNA and some proteins involved in the processes of translation, transcription, and replication have placed the notion of archaea on a firm footing [5-9]. Analysis of small subunit rRNA sequences revealed that there are two distinct phyla viz. euryarchaeota and crenarchaeota within this third domain [10]. Three more distinguished phyla viz. nanoarchaeota, korarchaeota and thaumarchaeota have later been introduced to the domain of archaea [11-17].

Subsequent work on archaea has revealed a lot of surprises that have invoked an urge in the scientific community to explore the world of these microbial life forms. Archaea have a unique mosaic combination of “eubacterial form and eukaryotic content”. Like bacteria, they are single- celled prokaryotes, devoid of nucleus or other cell organelles [18]. They usually share some major aspects of genome organisation and expression strategy such as presence of single circular chromosome and absence of introns, the operonic organisation of certain genes, presence of ribosomal-binding (Shine-Dalgarno) sites and so on; though there are some reports on the presence of archaeal introns [19,20]. Yet they possess a number of genes and metabolic pathways - especially the ones associated with the processes of transcription, translation and replication - typical of eukaryotes [21,22]. More than 30 ribosomal proteins are shared between the archaea and the eukarya that are not found in the bacteria. The structure of chromatin, presence of histones, significant similarity between proteins involved in information processing systems - all indicate a close evolutionary link between archaea and eukaryotes [23-26]. Archaea also possess some unique characteristics not shared by other domains. For example, their membrane is made of ether linked lipids. The glycerol phosphate backbone has got an opposite stereochemistry as compared to bacteria or eukaryotes [27,28]. They also exhibit some unique metabolism like methanogenesis and several unique enzymes e.g. specific types of DNA topoisomerases and DNA polymerases [29-31]. Till date there has been no report on archaeal virulence, but they have been found associated with the diseased state of colon and periodontal diseases [32,33].

Another intriguing feature of archaea is their unusual ability to survive and thrive in the extreme environmental conditions, such as in thermal vents, volcanic springs, hypersaline basins, alkaline lakes, acid mines, or even in petroleum deposits at deep underground which is completely devoid of oxygen. Furthermore, certain groups of archaea employ distinct strategies for energy conversion and hence, are characterised by special metabolic traits like methane production under anaerobic conditions, or sulphur respiration. Adaptation to such extreme environment or to atypical metabolism is expected to require special, adaptive gene and/or protein features - clearly distinguishable from those of the organisms living under the conventional ecological conditions. There are some reports on the molecular, physiological and evolutionary mechanisms of adaptation of some specific groups of extremophilic microbes, including some archaea, such as the organisms adapted to high temperature or salinity [34-39].

But to our knowledge, no comprehensive comparative study on lineage-specific and/or niche- specific genome/proteome features of the archaeal world has so far been reported.

Therefore, the domain archaea seems a deep sea where the researchers can dive into to get more and more information about their specific characteristics. Availability of complete genome sequences of hundreds of archaea has paved a way for comparative genomics and proteomics study. The lack of established model systems for large-scale experimentation on archaeal biology has made *in-silico* genome data mining even more crucial for archaeal genomics than they are in the cases of bacteria and eukaryotes. The present analysis offers the first large-scale comparative study of the proteomic architectures of all the archaeal species of publicly available genome sequences. Special emphasis has been given on the comparative analysis of methanogenic and sulphur metabolising archaea with an aim to unveil the special niche-specific molecular features, if any, of these two groups of microbes with specialised life-style. Identification of such features may not only give an insight into the molecular mechanism of ecological adaptation in archaea, but may also be important from the metagenomic or biotechnological view-points.

## Results and discussion

### Analysis of the whole archaeal dataset

#### Amino acid usage profile within the groups

**Figure 1:**
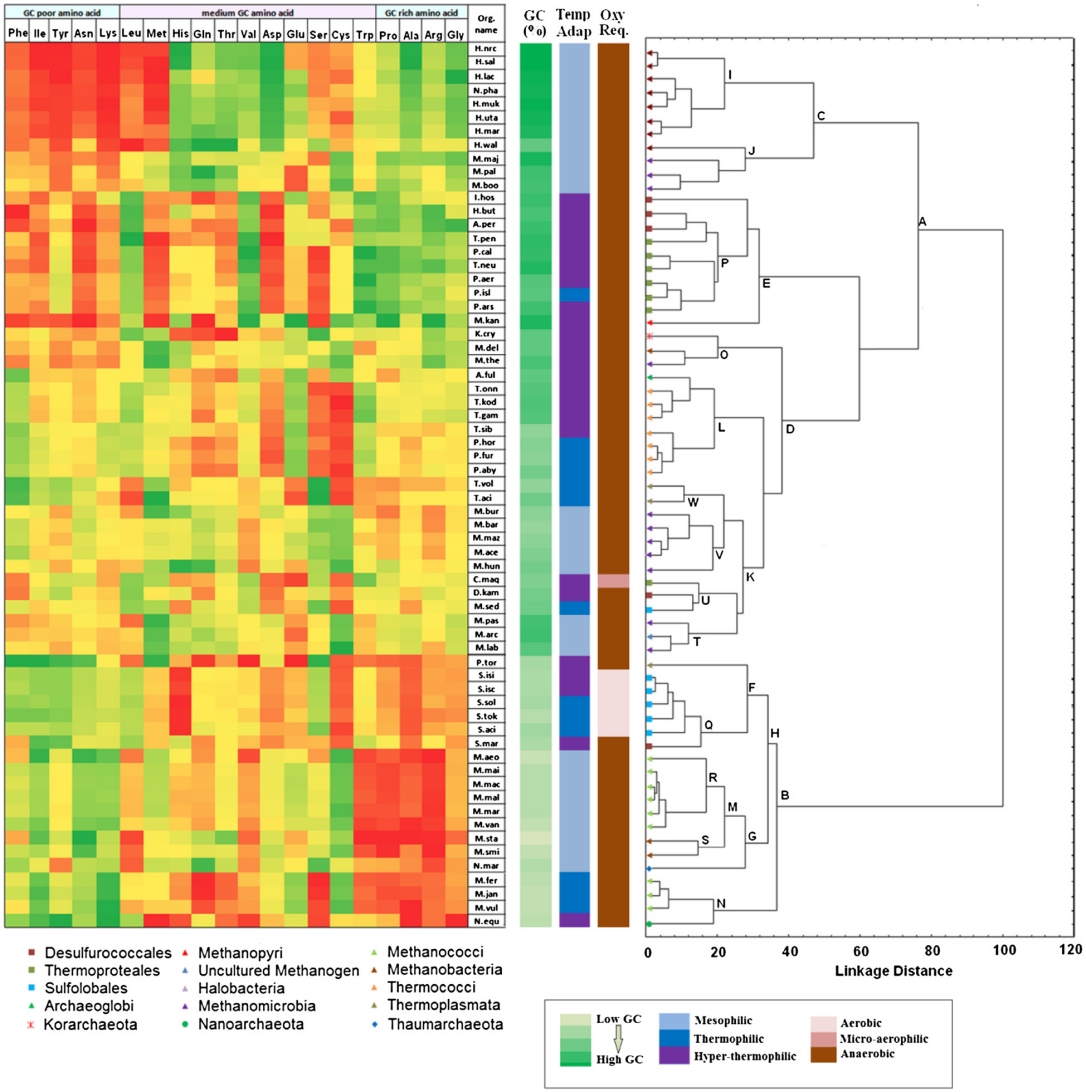
**Grouping of archaea according to their standardised amino acid usage.** The left panel is a pictorial representation of relative amino acid usage in the respective archaea. The over- representation and underrepresentation of amino acid residues in the organisms are shown in green and red colored blocks, respectively. The right panel depicts the complete linkage clustering on the Euclidean distances between the relative abundances of different amino acid residues in the encoded proteins of organisms. Organism abbreviations are listed in Additional file 1. The panel in between shows the short names of the archaea, their genomic GC content, temperature adaptation and oxygen requirements respectively.

Figure 1 depicts the complete linkage clustering of 69 archaeal organisms under study on the basis of the average frequencies of occurrences of different amino acid residues in the respective proteomes (for organism details, see Additional file 1). The left panel of the figure represents a heat map of the relative amino acid usage values, where the colour gradient from red to green shows the increment in the respective values in a particular column i.e. for a particular amino acid residue. The residues like Tyr, Lys, Asn, Ile and Phe, encoded with AU-rich codons, are presented in the extreme left side of the heat map, while the residues encoded by GC-rich codons, such as Pro, Ala, Arg and Gly are displayed in the extreme right side of the map. The right panel of Figure.1 represents the clustering of the organisms on the basis of their amino acid usage, where the organisms are segregated in two distinct sub-clusters under the nodes A and B. The organisms clustered under the node A, in most cases, have an average genomic GC-content ≥ 40%, while those segregated under the node B are marked by relatively less G + C-bias (average GC-content ≤ 40%), indicating that the amino acid usage in the archaeal species under examination are primarily governed by their genomic G + C-bias, though certain lineage-specific and/or niche-specific trends in their amino acid usage can also be identified within or even across different clusters. Appearance of red coloured blocks at two diagonally opposite corners of Figure 1, indicating under-representation of the residues encoded by AU-rich codons in organisms with high GC-content and of those encoded by GC-rich codons in GC-poor organisms, also advocates for influence of genomic GC-bias on archaeal proteome composition.

However, a closer look at the heat map reveals that the amino acid preferences by archaeal species are not solely governed by the mutational bias of the respective genomes. Taxonomic or ecological background of the species may also play important roles in shaping their protein composition. In many cases, members of the same phyla, class or order appear under distinct nodes far apart from one another, yet they share some common compositional features, which may not always comply with their genomic GC-bias. For instance, *P. torridus* (P. tor), a thermoplasmata species, appears under node F, far apart from *T. acidophilum* (T.aci) and *T. volcanium* (T. vol), two other members of thermoplasmata that clustered together under node W.

But in all these species, the usages of Phe and Met, two residues encoded by AU-rich codons, are higher than sulfolobales, methanococci or nanoarchaeota– the genomic GC-contents of which are comparable to or lower than those of thermoplasmata. These three species are also typified by relatively low usage of Glu, Leu and Cys and higher usage of Ser. The sulfolobales having similar GC-bias as P. tor (35-37%) in general, segregate together with P. tor under node F, but they differ in the usage patterns of amany of the residues like Leu, His, Asp, Trp etc., and ma ny of these features are also shared by *M. sedula* (M. Sed) - the only member of sulfolobales in the dataset with much higher GC-content (46%, under node U). A trend of lower usage of Asp and Met and higher usage of Leu and Val is observed in all thermoprot eales including *C. maquilingensis* (C. maq) with much lower GC-content (43%), which has clustered together with M. sed and D. kam - far apart from other thermoproteales (GC-content >50%). Each of the three single-member phyla in the dataset, namely nanoarchaeota, korarchaeota and thaumarchaeota, exhibit distinct trends in amino acid usage and appear as singular species in separate branches under the nodes N, O and G respectively. A detail examination of the biological implications of such conspicuous amino acid usage patterns is, however, beyond the scope of the present study and will be taken up separately in future.

From their proteome compositional features, archaea appear to adapt to specific niche or life- style. Most of the methanogens exhibit relatively high frequencies of Cys. All the halophiles have clustered under node I (except H.wal) and are marked with high usage of Asp, Thr and His and low occurrence of Cys, Leu and Met. It is worth noting that *H. walsbyi* (H. wal) (displayed under node J) has much lower GC-content (48%) than other halophiles (> 60%), yet it shares many typical features of high salt-adapted proteomes like under representation of Lys, Phe, Tyr, Met and Leu (all of these except Leu are encoded by AU-rich codons), and over representation of Asp, Thr, His etc. It may, therefore, be said that the genomic GC-bias, taxonomic history and life-style or niche adaptation – all have played important roles in sculpting the amino acid composition of an archaeon.

#### Physico-chemical characteristics of the proteomes of different groups of archaea

**Figure 2:**
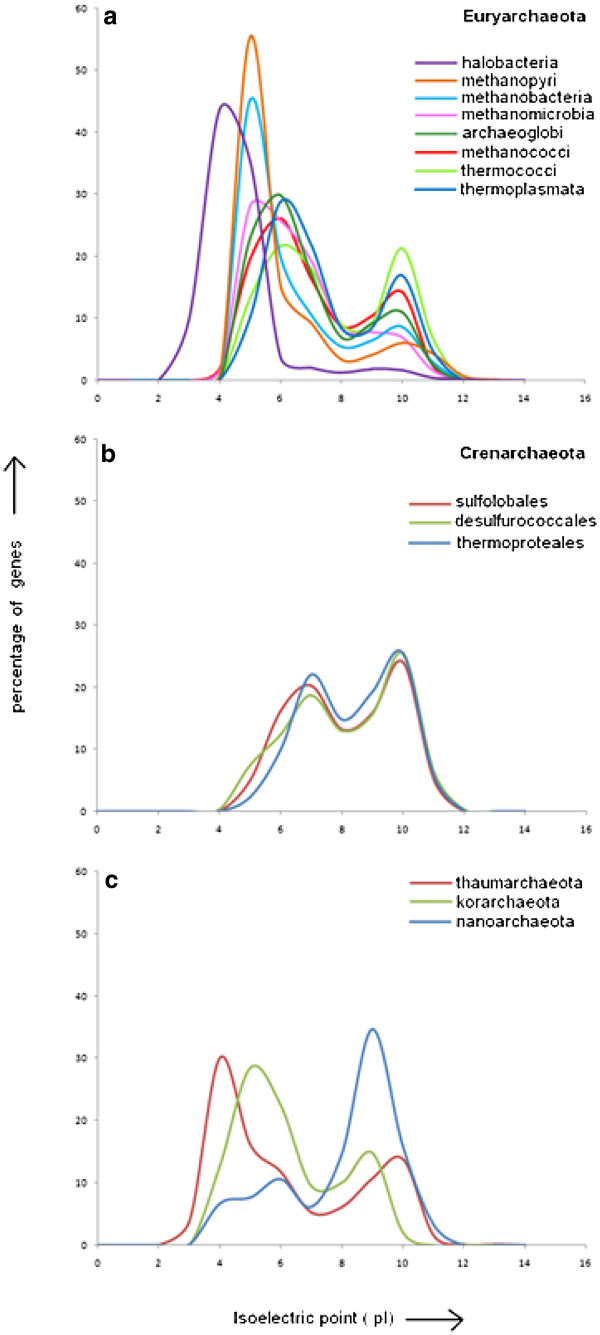
Isoelectric point distribution patterns in a) euryarchaeal classes b) crenarchaeal orders and c) the rest three phyla of archaea.

With a view to understand the physico-chemical basis of distinct trends in amino acid composition of archaeal proteomes within and across different lineages, various physico - chemical parameters like mean hydropathy, aromaticity and isoelectric points have been calculated individually for all predicted protein sequences for each of the organisms under study. While the distribution of mean hydropathy and aromaticity values show little variations across the proteomes (data not shown), significant variations have been observed among different groups of archaeal proteomes in distribution of pI or isoelectric points of predicted proteins. Figure 2 shows the average distribution profiles of the predicted isoelectric points (pI) of the deduced amino acid sequences for different classes/orders of archaeal organisms under study.

We have categorised the organisms according to their classes in case of euryarchaeota (Figure 2a) and down to their orders in case of crenarchaeota (Figure 2b), as the entire crenarchaeal group comes under a single class viz. thermoprotei. The remaining three phyla viz. korarchaeota, nanoarchaeota and thaumarchaeota (Figure 2c), have only one fully sequenced organism in each case, so there is no need of any further division.

In most of the cases, bimodal distributions of isoelectric points are observed with an acidic peak at pI range of 5.0–5.5 and a basic peak at ~9.5 [Here, “acidic peak” refers to the frequency peak in the isoelectric point plot, where the pI range lies around the acidic pH region < pH 7.0, similarly, “basic peak” refers to the region around pH >7.0]. Being the largest phylum in the archaeal world, euryarchaeota consists of eight classes and for all these classes except thermococci, the acidic peak is significantly higher than the basic peak, implying the overall acidic nature of the euryarchaeal proteomes, irrespective of their genomic GC-bias or niche adaptation. Among these, Halobacteria, a group of halophilic archaea, has the most acidic proteome showing a large acidic peak around pI 4.0 and almost no peak at basic pI – a feature attributable to over representation of Asp and under representation of Lys, as observed earlier in most of the microbial halophiles [37,40]. The only methanopyri in the dataset, *M. kandleri*, which is known to have dual adaptation to high salinity and high temperature, also exhibits a large and sharp acidic peak around pI 5.0 along with a small basic peak. A large acidic peak at pI 5.0 is also displayed by methanobacteria. Though their salinity adaptation is not yet reported, they have been found in large amounts in the tropical estuarine sediments along with other halophilies [41]. For all other euryarchaeal proteomes, acidic peaks (at pI values around 6.0) are slightly larger than the respective basic peaks (at pI values around 10.0), implying that these proteomes are also comparatively acidic in nature, whereas thermococci stands out as an exception, probably owing to their adaptation to high temperature and sulphur metabolism.

Crenarchaeal organisms are all under one class viz. thermoprotei. Though we have further divided them into three orders, they do not exhibit any significant variation in their pI profiles. For all three orders, proteomes are comparatively basic in nature (Figure 2b).

As reported earlier, nanoarchaeota, being a parasitic-hyperthermophile, has a highly basic proteome, while the proteome of thaumarchaeota, being mesophilic in nature, is comparatively acidic (Figure 2c) [12,34]. Korarchaeota, in spite of being a hyperthermophile, has an acidic proteome, which is quite surprising in view of earlier reports on thermal adaptation of microbial proteomes [42]. However, considering the fact that korarchaeal samples were collected from the Obsidian Pool, Yellowstone National Park [13], the possibility of hypersaline adaptation in *K. cryptofilum* cannot be ruled out and in that case, the halophilic signatures of its proteome may overshadow the thermophilic characteristics, as observed in *M.kandleri.*

#### Distribution of cluster of orthologous groups of proteins

**Figure 3:**
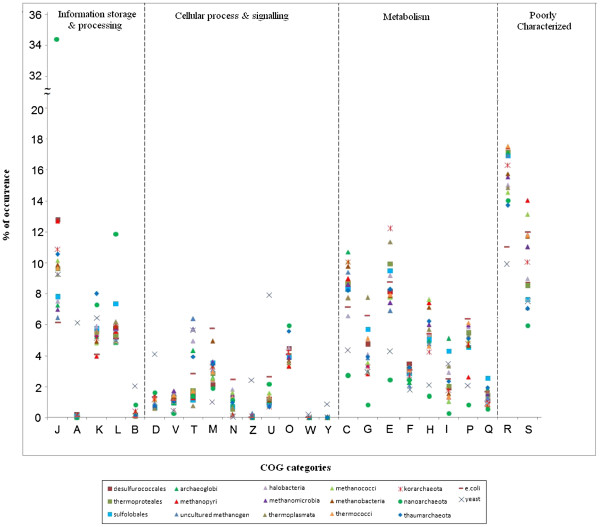
Distribution of COGs categories of the entire archaeal group under study and their comparison with E.coli (member from bacterial kingdom) and yeast (member from eukaryotic kingdom).

With a view to assess the similarities and divergences in the genetic make-up of different classes/orders of archaeal species, we took advantage of the COGs functional classification, which is based on orthologous relations among genes [43]. Figure 3 shows a plot of the ratio of normalised genes to total gene content for each COGs category in archaeal genomes of different taxonomic clases/orders, where *Escherichia coli* and *Saccharomyces cerevisiae* have been taken as control representatives of the bacteria and eukaryotic domains.

As revealed in Figure 3, the overall COGs distribution profiles of the archaeal groups are, in general, much closer to that of *E. coli* than to the Yeast profile. Majority of the COGs categories related to cellular processes & signaling are present in relatively low frequencies in archaea as compared to the eukaryotic representative *S. cerevisiae*. However, proteins belonging to the categories M (cell wall/memberane/envelope biogenesis) and N (cell motility) have higher frequencies in archaea as well as in *E. coli* than in yeast. The COGs related to metabolism also have in relatively high frequencies in archaea than in Yeast.

R & S, the two categories of poorly characterised genes, together encompass around 20 to 28% of predicted gene-products in each group of archaeal proteomes (Figure 3). Among the well characterised COGs categories, the ones showing the highest abundances in distribution profiles across different classes/orders are E (amino acid transport and metabolism), J (translation, ribosomal structure and biogenesis) and C (energy production and conversion), whereas the categories showing largest standard deviations are J (translation, ribosomal structure and biogenesis), T (signal transduction mechanism) and L (replication, recombination and repair). Nanoarchaeota shows a very different COGs distribution pattern with 34.5% of its total proteome falling under the translation, ribosomal structure and biogenesis (J) category ( Additional file 2). Strikingly enough, most of the COGs categories pertaining to metabolism, such as C, G, E, H, I, P and Q are significantly underrepresented in *N. equitans*. Such a conspicuous trend in COGs distribution in *N. equitans* may be attributed to the parasitic/symbiotic lifestyle of the organism. Methanopyri, the halophilic, thermophilic archaeon, is characterised with comparatively low amount of K (transcription) and P (inorganic ion transport & metabolism) category.

### Detail analysis of methanogenic and sulphur-metabolising archaea

The dataset of 69 archaeal species under study includes various types of extremophiles – the species thriving in extreme habitats such as thermal vents or hypersaline water as well as the species exhibiting specialised metabolism, such as methanogenesis or sulphur metabolism. Since the distinct genome/proteome features of thermophilic and halophilic organisms have been reported earlier [44,45], an attempt is made in the present study to delineate the niche-specific molecular features, if any, of the groups of archaea exhibiting specialised metabolic traits, i.e., the methane-producing archaea and the sulphur-oxidising/sulphur-reducing archaea (for details, see Additional file 3). It is worth mentioning at this point that these two groups of archaea also contain some thermophilic/hyperthermophilic and acidophilic organisms and as already mentioned, *M. kandleri* exhibits dual adaptation to thermophilic and halophilic environments [46].

#### Usage of amino acids with no GC bias at the codon levels

**Figure 4:**
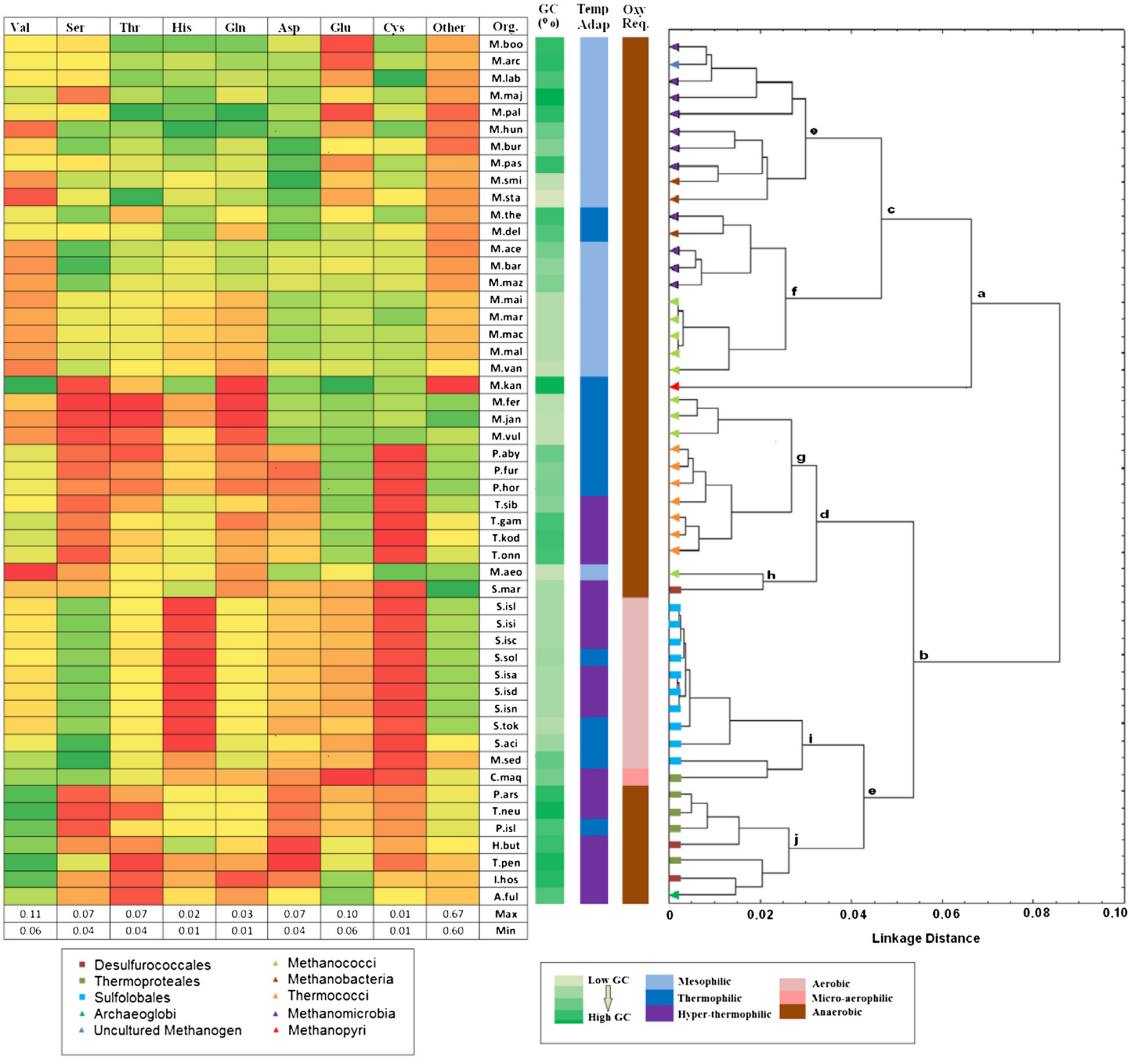
**heat map and clustering on the unbiased amino acid usage profile in the methanogenic and the sulphur metabolising group.** In the heat map, the column ‘other’ represents the sum of the frequencies of the rest of the amino acids i.e. those which are biased at their codon levels. The colour scheme and the clustering method is the same as described in Figure 1.

The trends in overall amino acid usage of methanogenic and sulphur-metabolising archaea have already been discussed in context of Figure 1, where the organisms are clustered according to their amino acid usage. But as depicted in this figure, the impact of genomic GC-bias on the amino acid usage is so strong that the organisms under study have been segregated, in effect, on the basis of their average genomic GC-content. However, one cannot rule out the possibility of existence of other selection pressures on amino acid usage, influence of which might have been overshadowed by the effect of the GC-bias and hence, could not be detected in Figure 1. In order to identify and characterise such selection pressures, if any, one needs to mask the pronounced effect of the genomic GC-bias in the cluster analysis and heat-map of amino acid usage. To this end, we present Figure 4, where despite of considering all the 20 amino acids, only those are chosen, which have no GC bias in their respective codons, viz. Val, Ser, Thr, His, Gln, Asp, Glu and Cys. The frequencies for the remaining amino acids are taken as a sum during the process of clustering, so that no artifact arises owing to this exercise.

As revealed in Figure 4, the two factors that primarily govern the usage of these eight amino acid residues are the oxygen requirement and temperature adaptation of the respective archaeal species. All aerobic species along with C. maq, the only micro-aerophilic archaeon in the dataset, are completely segregated from the anaerobic organisms and clustered exclusively under the node i, suggesting the significant influence of respiratory habits of the organisms on preferences for these amino acids. Dominance of temperature adaptation of the species is apparent from the observation that there are two major nodes a and b in Figure 4, dividing whole set of methanogens and sulphur-metabolisers under consideration into two major clusters, where all mesophiles except *M aeolicus* have been segregated under the node a, and all hyperthermophiles and majority of the thermophiles (except M. the, M. del and M. kan) have clustered together under the node b.

All crenarchaeal species except S. mar are clustered together under the node e. A carefu l examination of the accompanying heat map suggests that the unexpected segregation of S.mar and M.aeo apparently represents an artifact, since this segregation might be attributed to the similarity in the “others” column, which represents total frequencies of occurrence of the residues encoded by GC-rich/AU-rich codons.

In the heat map, all methanogenic archaea, in spite of their difference in genomic GC content and habitat, show an affinity for higher usage of Cys residues in their proteomes as compared to sulphur-metabolising counterparts. If we consider in terms of frequencies of occurrence, the Cys usages are almost double in case of methanogens [47]. Asp is more abundant in methanogens than the other group, whereas Leu is used more in sulphur metabolisers.

The highest and the lowest values of amino acids in the available orders of methanogens and sulphur metabolisers are presented in Table 1. Frequencies of certain residues, namely Ile, Asn, Lys, cys, Trp, Ala etc., exhibit two-to-three-fold variations within the dataset. Overall the proteomes of sulphur metabolisers are richer in aromatic amino acid residues, for example, Phe finds its highest value in thermococcales, Tyr is mostly used in sulfolobales and Trp has highest usage in thermoproteales.

**Table 1:** Highest and lowest values of amino acids in the methanogenic and sulphur metabolising orders

**Order with the highest value**	**Max value (%)**	**Amino acid**	**Min value (%)**	**Order with the lowest value**
Thermococcales	4.40	**Phe**	2.88	Methanopyrales
Methanococcales	9.92	**Ile**	4.81	Methanopyrales
Methanobacteriales	5.88	**Asn**	1.92	Methanopyrales
Methanococcales	9.49	**Lys**	3.99	Methanopyrales
Sulfolobales	4.74	**Tyr**	2.82	Methanopyrales
Methanocellales	3.00	**Met**	1.65	Methanopyrales
Desulfurococcales	10.97	**Leu**	8.65	Methanocellales
Methanopyrales	10.55	**Val**	6.81	Methanococcales
Methanosarcinales	6.83	**Ser**	4.64	Methanopyrales
Methanomicrobiales	6.07	**Thr**	4.48	Desulfurococcales
Methanomicrobiales	2.01	**His**	1.32	Sulfolobales
Methanomicrobiales	2.87	**Gln**	1.41	Methanopyrales
Methanobacteriales	6.18	**Asp**	4.39	Thermoproteales
Methanopyrales	9.99	**Glu**	6.18	Unclassified methanogen RC-I
Methanomicrobiales	1.34	**Cys**	0.56	Thermococcales
Thermoproteales	1.35	**Trp**	0.66	Methanococcales
Methanopyrales	8.33	**Arg**	3.35	Methanococcales
Methanopyrales	5.48	**Pro**	3.39	Methanococcales
Thermoproteales	9.40	**Ala**	5.55	Methanococcales
Methanopyrales	8.05	**Gly**	6.60	Sulfolobales

#### Correspondence analysis on amino acid usage with two groups

**Figure 5:**
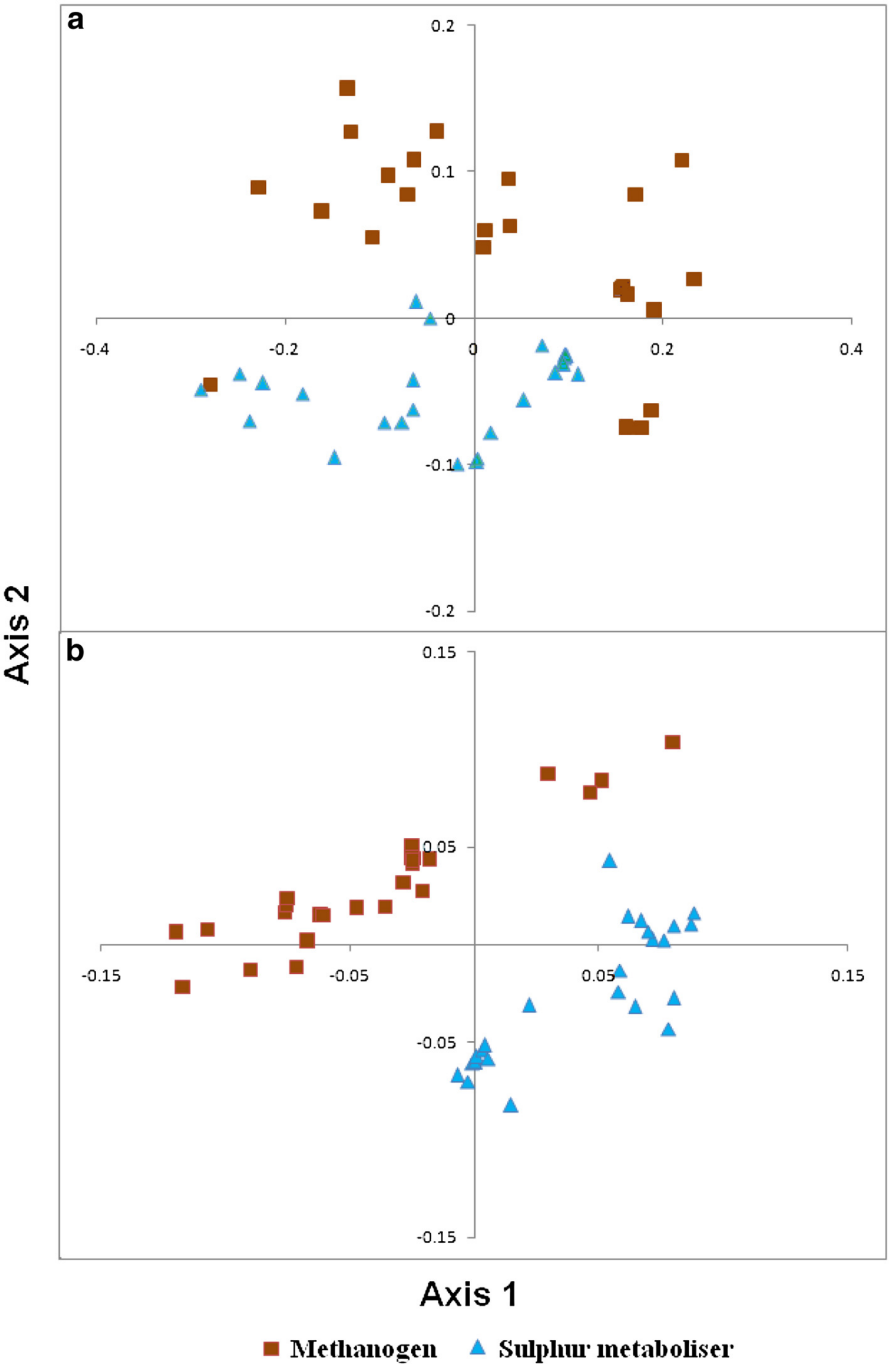
Plot of all the methanogens and the sulphur metabolisers in the plane of the first two axes generated by the correspondence analysis on a) all amino acid usage and b) unbiased amino acid usage.

Figure 5a represents the positions of all 51 organisms in the plane originated by the first two principal axes generated by Correspondence Analysis (COA) on all the amino acid usage. The first two axes explain 63.98% and 15.44% variability respectively, contributing a total of 79.42% to the total variation in the dataset. Unlike the cluster analysis, COA on amino acid usage segregates the two metabolising groups - the methanogenic and the sulphur metabolising organisms - along the second axis of the plane. There are, however, few exceptions. M.fer, M.jan and M.vul are placed in the fourth quadrant with other sulphur metabolisers and M.kan is placed in the third quadrant. *M. kandleri*, being a unique methane-producing archaeon known to date with dual adaptation to high temperature and high salinity, is marked with its conspicuous presence within the sulphur metabolising group instead of the group of methanogens [46,48]. A strong negative correlation between the first axis and the genomic GC content of the organisms have been observed with r = −0.97, p < 10–5, revealing the fact that the most promising variable contributing to the total variation in the entire dataset is the GC content of the organisms. The second axis shows a negative correlation with the isoelectric point of the proteomes with r = −0.71, p < 10^-5^.

Correspondence analysis on the unbiased amino acid usage dataset segregates the two types of organisms diagonally, i.e. the variable which decides the segregation should have almost similar correlation with both the axes (Figure 5b). The first two axes explain 63.47% of the total variation as a whole. Since the genomic GC-bias has hardly any influence on the usage of these amino acid residues, significant correlations of isoelectric point have been observed with both the axes(with r = 0.63 and −0.60 respectively).

#### Comparative analysis of physico-chemical features of proteomes

**Figure 6:**
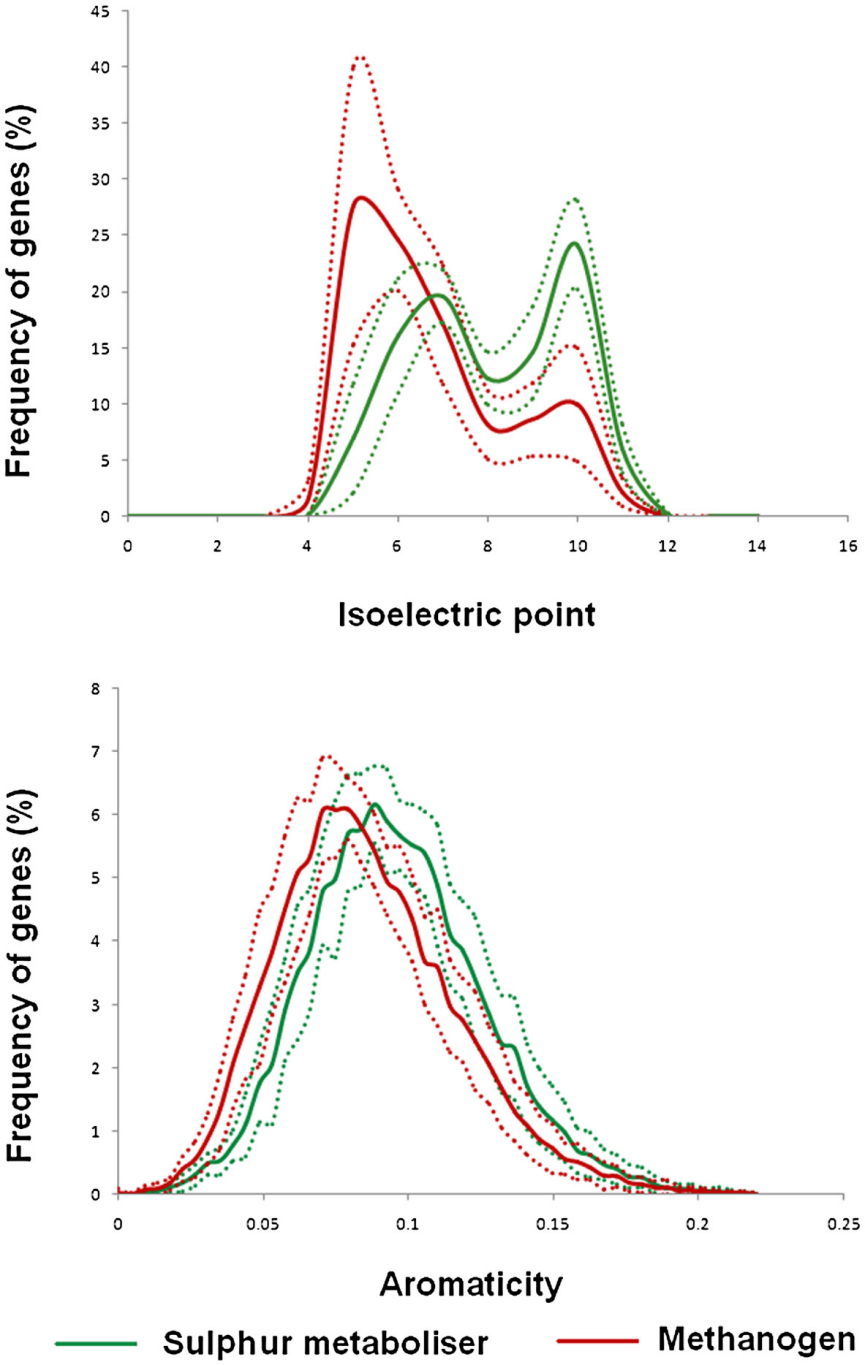
Isoelectric point and aromaticity distribution patterns in the two studied groups.

Among all the traditional amino acid usage indices variables, the isoelectric point distribution of the two groups show the most significant difference in them. Figure 6a shows the comparisons between the isoelectric point distributions of the two groups. Previously in Figure 2, the lineage-specific distribution of isoelectric points was presented, where distinct trends in pI profiles was observed in different archaeal phyla. But Figure 6a delineates the overall differences in pI profiles in two groups of proteomes of distinct metabolic traits, which reveals that methanogens, in general, have more acidic proteomes than their sulphur metabolising counterparts. The plot shows the average percentage number of genes ± the standard deviations in each pI range in both the cases. It is well established that thermophilic organisms are in general, characterised by basic proteomes [34,42,49]. Since all the sulphur metabolisers available to date are thermophilic/hyperthermophilic in nature, one could argue that the isoelectric point distribution of their proteomes is a mere reflection of their temperature adaptation. But if that was the case, we should have obtained basic proteomes in case of thermophilic/hyperthermophilic methanogenic organisms also, but this is not true. On the contrary, all the methanogenic organisms irrespective of their temperature adaptation individually show the trend of having acidic proteomes (data not shown). Methanogenic proteomes also exhibit relatively low usage of aromatic residues as compared to the sulphur-metabolising archaea (Figure 6b). This observation is in good agreement with the earlier proposition by Das et al. which suggested that higher occurrences of positively charged residues and aromatic residues may facilitate cation-pi interactions in thermophilic/hyper-thermophilic proteomes [34].

#### Surface charge distribution

**Figure 7:**
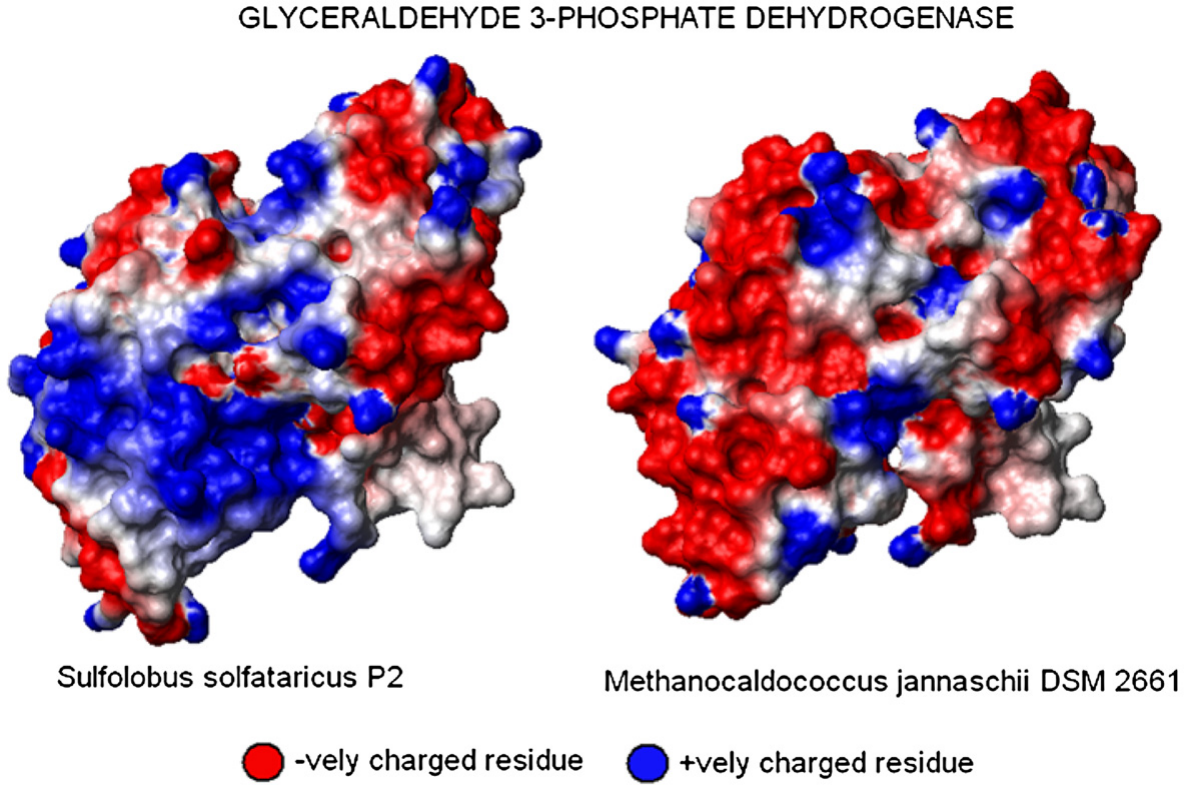
Surface charge distribution pattern of the protein glyceraldehydes-3-phosphate- dehydrogenase in M.jan (methanogen) and S.sol (sulphur metaboliser).

Since the distinct patterns in the average isoelectric point distribution in methano gens and sulphur-metabolisers points towards the overall acidic and basic natures of the proteomes of these two groups of archaea respectively, we further wanted to compare the surface charge distribution of orthologous proteins from these two groups. Figure 7 shows the surface charge distribution of the protein glyceraldehyde-3-phosphate-dehydrogenase, from two different organisms, *S.solfataricus P2* as a representative from sulphur metabolising group and *M. jannaschii DSM 2661* from methanogenic group, with sequence similarity of 67%. To avoid the effect of thermal adaptation on the surface charge distribution patterns, both of these thermophilic organisms are consciously selected. Genomic GC contents are also quite similar in both of these organisms. The selection of this particular protein is justified by the fact that it is the only protein whose crystal structures are available for both of these groups [50,51]. The surface charge view has been generated by the program MOLMOL [52], as described in the Materials & Methods section. Both the organisms are hyperthermophilic and hence should have positive charges on the surfaces, as reported earlier for proteins from other hyperthermophilic microbes [34,42,49]. But as can be seen from Figure 7, the protein from *M. jannaschii DSM 2661* contains more negatively charged residues on its surface, whereas its *S.solfataricus P2* ortholog have more positively charged residues on the surface. This observation re-confirms the findings on the isoelectric point distribution in two groups of organisms under study.

#### Amino acid substitution in orthologous sequences

Since comparative studies on isoelectric po int distribution and surface charge distribution in proteins of methanogenic and sulphur-metabolising archaea have revealed a clear trend in higher usage of acidic residues in the former group of species (Figure 6 &7), it is tempting to examine the general trend, if any, in the amino acid substitution patterns between the orthologous proteins from members of these two different groups. Among all the organisms chosen for this study, we selected two organisms from two groups such that their thermal adaptations, genomic GC contents, genome sizes, and the phylum they are placed in are identical. M.del and T.onn suited well ( Additional file 3) for analysing amino acid substitution in their orthologous sequences.

Thus, a distinct trend in the resultant substitution patterns across the orthologs from these organisms can be attributed to the differences in their metabolic traits. The amino acid sequences of these orthologous genes were aligned using ClustalW and the amino acid replacements are arranged in a 20 × 20 matrix using Substitution Pattern Analysis Software Tool (SPAST), a program in C++, developed in-house [53]. Frequencies of all possible amino acid replacements (i.e. (20 × 19)/2 = 190 possible pairs of replacements) between the orthologous protein sequences were determined in the direction from the methanogenic archaea M.del to the sulphur metabolising archaea T.onn, following the method reported by Paul et al., described in details in the Materials & Methods section [37].

The prominent trends observed in such replacements are as follows: i) Cyst ines of M. del proteins are replaced by various residues in their T. onn orthologs, ii) different residues of M.del proteins are substituted by aromatic residues, especially by Trp in T. onn orthologs, iii) Ser and Asp of M.del sequences are replaced by other residues in T. onn sequences. T.onn proteins also show a tendency of acquiring Lys in lieu of other residues. Thus, there is a prevale nce of overall gain in Lys as well as in aromatic residues, especially Trp, and loss in Cys, Ser and Asp in the sulphur-metaboliser T. onn, as compared to the methanogen M.del. A careful scrutiny of the left panel of Table 2 also reveals that the T. onn proteins show a trend of gaining charged residues, not only the positively charged Lys, but also the negatively charged residue Glu in place of uncharged residues of their M.del orthologs.

**Table 2:** Top 20 amino acid pairs displaying highest bias in terms of differences and ratios in number of forward (methanogens → sulphur metaboliser) and reverse (sulphur metaboliser → methanogen) replacements in 213 orthologous proteins from M.del to T. onn

**Amino acid replacements between 213 orthologous proteins of M.del & T. onn**									
**Most biased in gain**					**Most biased in ratio**				
**Pair**	**Ratio**	**Fwd. No**	**Rev. No.**	**Gain**	**Pair**	**Ratio**	**Fwd. No.**	**Rev. No.**	**Gain**
R → K	1.97^##^	871	443	428	Q → W	8.00*	8	1	7
E → K	2.03^##^	615	303	312	C → M	7.50**	15	2	13
D → E	1.58^##^	723	458	265	H → W	6.00**	18	3	15
S → K	3.91^##^	274	70	204	C → I	6.00^##^	30	5	25
D → K	2.84^##^	281	99	182	C → S	4.75^##^	38	8	30
S → A	1.65^##^	442	268	174	C → N	4.67**	14	3	11
S → E	2.41^##^	296	123	173	D → W	4.33*	13	3	10
I → L	1.20^#^	838	701	137	R → W	4.25^##^	34	8	26
R → E	1.40^##^	436	312	124	S → K	3.91^##^	274	70	204
M → L	1.45^##^	379	262	117	C → A	3.83^##^	92	24	68
G → K	2.22^##^	182	82	100	M → W	3.60**	18	5	13
T → V	1.48^##^	275	186	89	C → V	3.55^##^	71	20	51
A → K	1.77^##^	188	106	82	C → G	3.50**	21	6	15
L → F	1.30**	328	252	76	S → W	3.50*	14	4	10
M → I	1.51^##^	204	135	69	D → Y	3.24^##^	55	17	38
C → A	3.83^##^	92	24	68	C → T	3.20#	32	10	22
S → T	1.33**	275	207	68	C → F	3.17**	19	6	13
D → N	1.43#	216	151	65	D → K	2.84^##^	281	99	182
T → L	1.62^##^	165	102	63	V → W	2.70**	27	10	17
H → Y	2.21^##^	106	48	58	I → W	2.44*	22	9	13

* significant at *p* < 0.05, ** significant at p < 0.01, ^#^ significant at *p* <0.001, ^##^ significant at p < 0.0001.

#### COGs category distribution in the two groups

**Figure 8:**
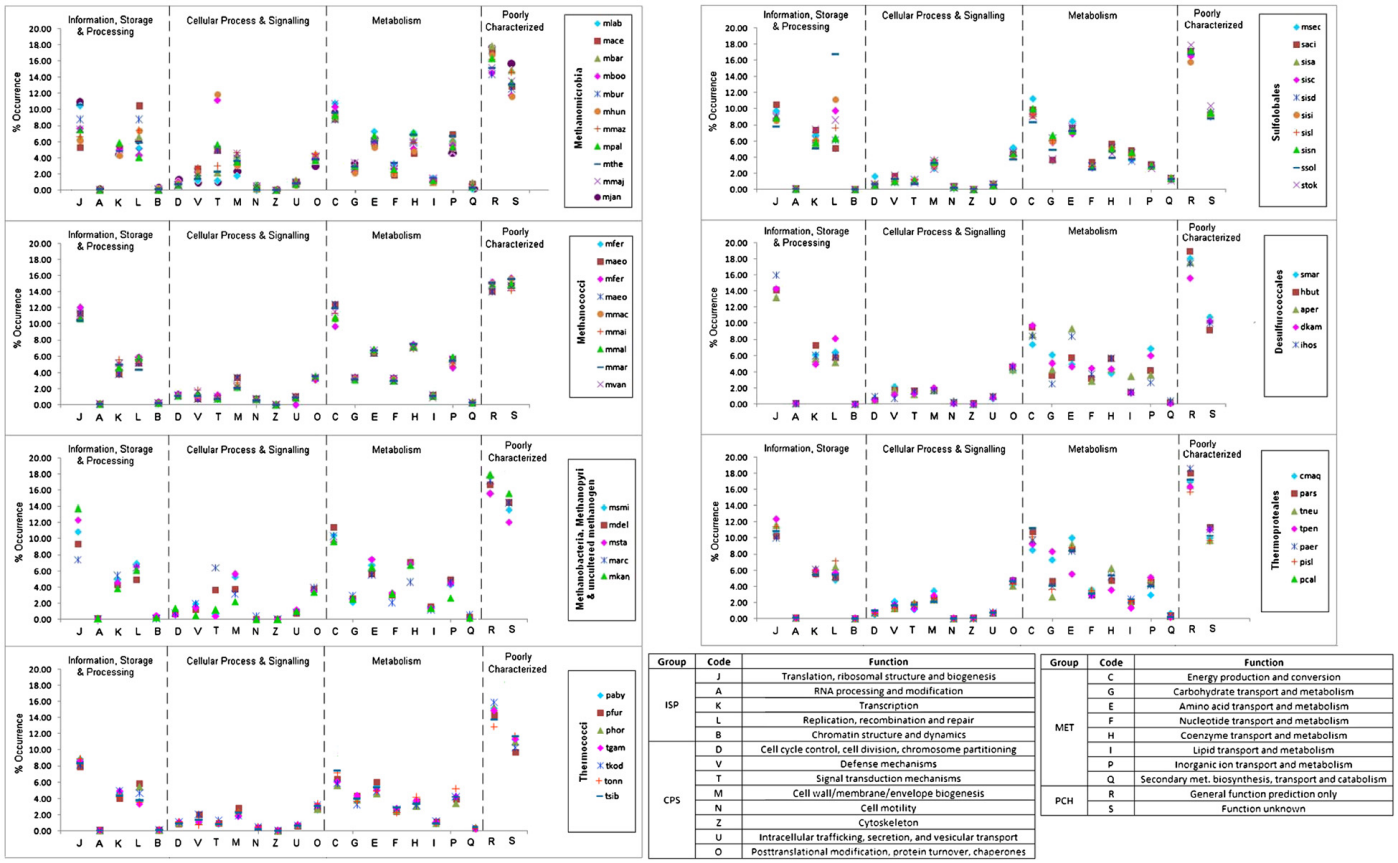
Plot of percentage occurrence of COGs categories in different groups of methanogens and sulphur metabolisers.

The average COGs distribution profiles of different orders/classes of the archaeal domain, along with those of *E. coli* and yeast, have already been depicted in Figure 3. Here we intend to examine the distribution of different functional COGs categories at the level of individual proteomes of the methanogenic and the sulphur metabolising groups of archaea. Wide variations in COGs distribution patterns have been observed among members of methanomicrobia and sulfolobales. Appreciable intra-class/order variations have also been observed among the members of methanobacteria, desulfurococcales and thermoproteales for certain COGs categories, while for methanococci and thermococci, such variations are not so apparent (Figure 8).

Among different COGs categories, certain categories like J (translation, ribosomal structure & biogenesis), L (replication, recombination & repair), M (cell wall/membrane biogenesis), C(energy production & conversion) etc. exhibit more intra-species divergences within a specific class/order, while the categories like U (intracellular trafficking, secretion & vesicular transport), V (defence mechanism), O(post translational modifications, protein turnover, chaperons) or Q (secondary metabolite biosynthesis, transport and catabolism) show little var iation within or even across different taxonomic orders. Interestingly enough, all three orders under the class thermoproteales of crenaerchaeota, namely sulfolobales, desulfurococcales and thermoproteales exhibit, in general, appreciable intra-order inter-species variations in frequencies of occurrences for most of the COGs categories pertaining to metabolism, but not for the COGs categories under Cellular Processes & Signaling. Such intra-class variations in frequencies are relatively less, in general, in cases of euryarchaeal organisms, both for metabolism and cellular processes & signaling COGs categories (Figure 8, left panel).

#### Identification of core, but exclusive COGs of methanogens

The next objective of this endeavour is to identify the niche-specific COGs, if any, in two metabolic groups of archaea under study. To this end, all annotated COGs IDs from the 25 methanogenic archaea of the current dataset are enlisted and searched against one another to get the core methanogenic COGs IDs. Each of these core COGs are then searched against the individual COGs contents of 26 sulphur metabolising archaea under examination. In this way we have identified 22 unique COGs, which are present in all methanogens, but not in any of the sulphur-metabolisers of the dataset. A further search confirmed that these 22 COGs are not present in any other archaeal proteome under study. Table 3 enlists these methanogen-specific proteins as well as the domains found in their sequences. A reverse search for COGs present in all sulphur-metabolising species, but not in any of the methanogens, does not yield any exclusive protein.

**Table 3:** Methanogen-specific COGs ID and their descriptions

**COG ID**	**Description**	**Domains found on TIGR**	**Domains found on Pfam**
COG4058	Methyl coenzyme M reductase, alpha subunit	met-coenzyme M reductase	met-coenzyme M reductase
COG4054	Methyl coenzyme M reductase, beta subunit		
COG4056	Methyl coenzyme M reductase, subunit C		
COG4055	Methyl coenzyme M reductase, subunit D		
COG2710	Nitrogenase molybdenum-iron protein, alpha and beta chains	methanogenesis marker protein 13	oxidored-nitr
COG1348	Nitrogenase subunit NifH (ATPase)	NifH	Fer4-NifH
COG4008	Predicted metal-binding transcription factor	methanogenesis marker protein 9	Not found
COG4070	Predicted peptidyl-prolyl cis-trans isomerase (rotamase), cyclophilin family	methanogenesis marker protein 3	
COG4002	Predicted phosphotransacetylase	methanogenesis marker protein 4	
COG4032	Predicted thiamine-pyrophosphate-binding protein	sulfopyruvate decarboxylase	TPP enzyme
COG2144	Selenophosphate synthetase-related proteins	methanogenesis marker protein 2	AIRS
COG4063	Tetrahydromethanopterin S-methyltransferase, subunit A	Methyl transferase (Mtr)	Methyl transferase (Mtr)
COG4062	Tetrahydromethanopterin S-methyltransferase, subunit B		
COG4061	Tetrahydromethanopterin S-methyltransferase, subunit C		
COG4060	Tetrahydromethanopterin S-methyltransferase, subunit D		
COG4059	Tetrahydromethanopterin S-methyltransferase, subunit E		
COG4050	Uncharacterized protein conserved in archaea	methanogenesis marker protein 5	Domain of unknown function (DUF)
COG4051	Uncharacterized protein conserved in archaea	methanogenesis marker protein 17	
COG4065	Uncharacterized protein conserved in archaea	methanogenesis marker protein 14	
COG4014	Uncharacterized protein conserved in archaea	***	
COG4029	Uncharacterized protein conserved in archaea	methanogenesis marker protein 6	
COG4052	Uncharacterized protein related to methyl coenzyme M reductase subunit C	methanogenesis marker protein 7	

#### Estimation of COGs shared mutually between distinct groups of methanogens and sulphur metaboliser

**Figure 9:**
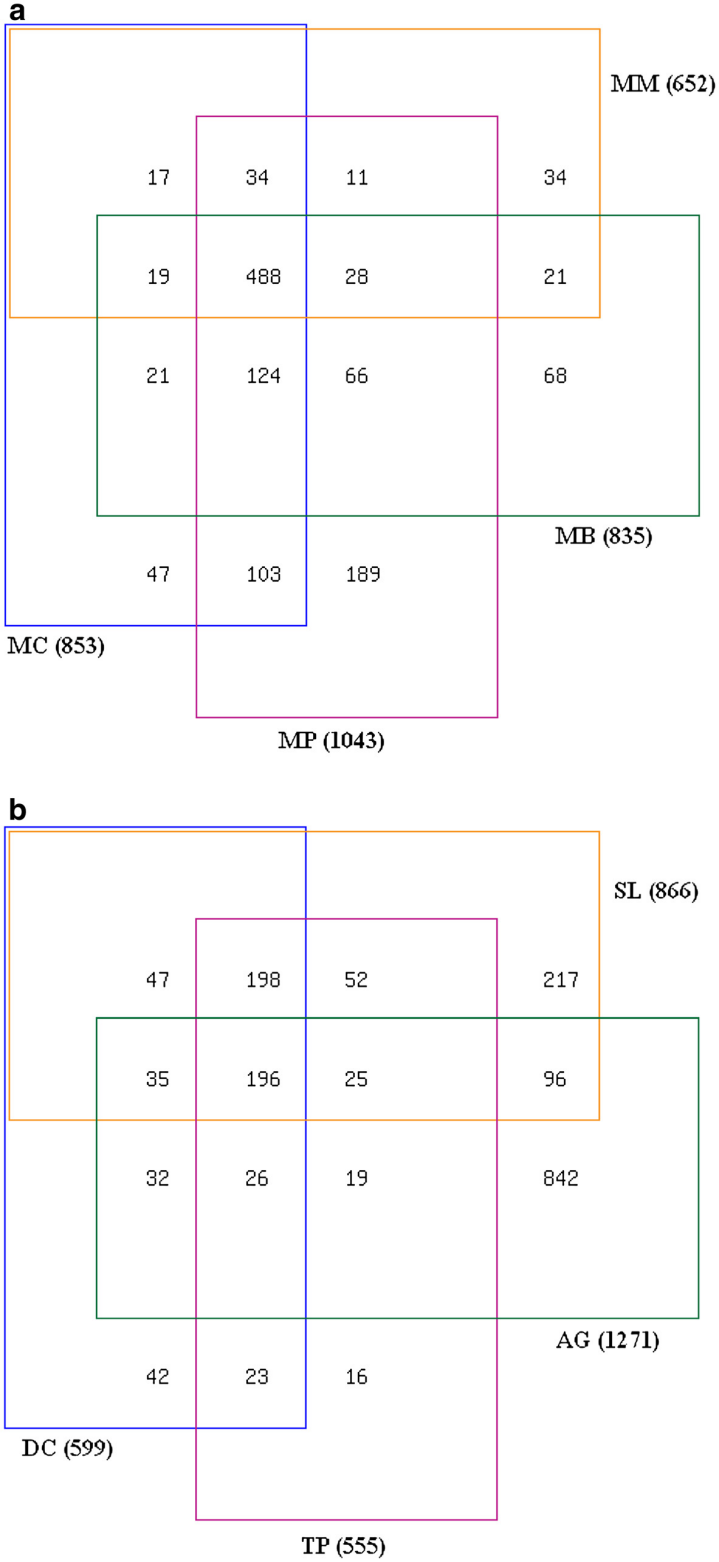
**Four-variable venn diagram of the COGs possession in a) four methanogenic classes and b) four sulphur metabolising orders.** MM- methanomicrobia, MB- Methanobacteria, MP- Methanopyri, MC- methanococci, SL-Sulfolobales, AG-Archaealglobales, TP-Thermoproteales, DC- Desulfurococcales.

In order to understand the evolutionary relationships between different classes of methanogens and different orders of sulphur metabolisers, the distribution of COGs in those groups of archaea have been examined. Figure 9a &9b depict four-variable venn diagrams for the respective groups of methanogens and sulphur metabolisers. The total number of COGs IDs for each group is given within a parenthesis. In case of methanogenic group, the number of exclusive COGs for the class methanococci, methanopyri, methanobacteria and methanomicrobia are 47, 189, 68 and 34 respectively. For the other group, the numbers for the order desulfurococcales, thermoproteales, archaeoglobales and sulfolobales are 42, 16, 842 and 217 respectively. All the methanogenic groups contain a core group of 488 COGs, whereas all the sulphur metabolisers share a core group of 196 COGs ( Additional file 4). A further investigation into the categories of the core COGs revealed that apart from J, R and S, methanogens have E and H, whereas sulphur counterparts have K and L respectively among their top five COGs categories (for COGs category, see Additional file 2). So the methanogens have more number of common COGs of the group metabolism and the sulphur counterparts have more number of information storage and processing COGs common in them.

## Conclusion

The present study gives an account of amino acid usage, physico-chemical features and COG repertoire of 69 archaeal species of varying GC-content, habitats, respiratory habits and metabolism. Amino acid usage pattern in archaea, in general, is dominated by the genomic GC- content, but in some cases niche-specialisation overrules the GC-bias. For amino acids having no GC-bias at their codon levels, environmental factors like oxygen requirement or temperature adaptation appear to be the primary selection forces. Among the physico-chemical parameters, the overall charge profile and aromaticity of proteins seem to modulate or be modulated by the metabolic traits and/or niche adaptation of the respective species. All methanogenic proteomes, irrespective of their temperature or salinity adptation, are relatively acidic and have higher usage of Cys, while the proteomes of sulphur metabolisers are more basic and aromatic in nature. The atypical (acidic) nature of the thermophilic archaeon *K. cryptofilum* is surprising and demands further investigation in future. So far as COGs repertoire is concerned, crenarchaeal organisms display higher intra-order variations as compared to euryarchaeal counterparts, especially for the proteins involved in metabolism, probably because the divergence of sulphur reduction pathways from those of sulphur oxidation. There are 22 COGs, which are found in all methanogenic archaea under study, not in any other archaea. No such core COGs could be found exclusively within sulphur-metabolising groups.

Identification of distinct trends in amino acid usage, physicochemical properties and COG distribution profiles in methanogens and sulphur-metabolisers, aerobic and anaerobic archaea or korarchaeota and nanoarchaeota point towards the diverse evolutionary strategies for niche specialisation in the archaeal world. Characterisation of such niche-specific features may have far-reaching implications of metagenomic or biotechnological perspectives.

## Method

### Sequence retrieval

The complete genome sequences and the predicted protein coding sequences of 69 (all the fully sequenced archaea available by the year 2009) archaea have been downloaded from NCBI GenBank. In order to reduce the sampling errors, the annotated ORFs having less than 100 codons in every genome have been excluded from the analysis. Additional file 1 and Additional file 3 show the basic information about all the archaea under study and about the two studied groups.

### Amino acid usage

Relative amino acid usage frequencies for each organism have been calculated from CODONW [54]. Heat map represents the pictorial version of amino acid frequencies all the organisms, where the colour gradient from red to green in every column shows the increasing values of abundance for a particular amino acid.

### Cluster analysis and correspondence analysis on amino acid usage

To find out the inter-proteomic differences between organisms, the correspondence analysis and the cluster analysis on amino acid composition are carried out using STATISTICA (version 6.0) for all organisms [55]. Correspondence analysis has been done for the two groups of organisms viz. sulphur metabolisers and methanogens. This analysis generates a series of orthogonal axes with each subsequent axis explaining decreasing amount of contribution to the total variation in the dataset.

### Indices used to identify the amino acid usage pattern

To identify the major factors influencing the amino acid usage we calculated the average hydrophobicity (Gravy Score), aromaticity, aliphatic index, instability index and isoelectric point distribution for every organism. We observed significant variation of isoelectric point distribution in case of all the organisms and aromaticity distribution pattern along with the pI distribution in case of the two studied groups, hence we present here the same.

### Surface charge distribution

The surface charge distributions are mapped onto the predicted surface using the program MOLMOL [52]. The protein used here is glyceraldehyde-3-phosphate-dehydrogenase from both the methanogenic and the sulphur metabolising archaea viz. *M. jannaschii DSM 2661* and *S.solfataricus P2* respectively (PDB ID 2YYY and 1B7G)*.*

### Amino acid exchange bias with orthologous sequences

Orthologous sequences between M.del and T.onn are taken using the tBlastx program [56]. Orthologs are defined as those with more than or equal to 40% similarities and less than 20% difference in length and e value ≤ 1E-10. The amino acid sequences of 213 orthologous genes are aligned using the pairwise alignment program ClustalW [57] and the amino acid replacements are obtained in the form of a matrix, using a program developed in-house in Visual Basic [53]. Under unbiased conditions, the ratio of forward to reverse substitutions is expected to be 1:1 for each pair of residues. To test this hypothesis, the observed and expected numbers (based on a 1:1 ratio) are recorded for each pair of residues and the chi-square test is applied to assess the significance of the directional bias, if any, at significance levels of 10–3 to 10–6. For a given pair of amino acids, the ‘forward’ direction exhibited the more common of the two replacements in the conversion of methanogenic to the sulphur metabolising proteins. To assess the significance of the directional bias, if any, replacement values are compared by 2 × 2 contingency tables having one degree of freedom. For each pair of replacements, the first and second rows of the contingency table represented the number of replacements from one particular residue (say, i) to another (say, j) of the pair and the total count of the remaining replacements (say, k) from the residue i (where k ≠ j) respectively.

### COGs (cluster of orthologous groups of proteins) distribution

The predicted COGs annotations for each protein of all the organisms have been done with the help of WebMGA server [58]. Only those proteins are taken for which COGs IDs have been annotated and hence other proteins are excluded. The percentage number of COGs category present in every organism has been calculated. For a better resolution of the gene possession of every phylum, the methanogens and sulphur metabolisers have been further divided into their corresponding classes and orders respectively.

## Abbreviations

COGs, Cluster of Orthologous Group of proteins; COA, Correspondence Analysis; SPAST, Substitution Pattern Analysis Software Tool.

## Competing interests

The authors declare that they have no competing interests.

## Authors’ contribution

ARC contributed to the development of work plan, carried out sequence analysis and other statistical/computational studies and drafted the manuscript. CD conceived the work, participated in the design and coordination of the study and revised the manuscript critically for important intellectual content. Both the authors read and approved the final manuscript.

## Supplementary Material

Additional data file1 Detail information about all archaea under study. Click here for file

Additional data file2 Details of COGs category. Click here for file

Additional data file3 Details of all sulphur metabolising and methanogenic archaea under study. Click here for file

Additional data file4 Descriptions about the methanogenic and sulphur metabolising core COGs. Click here for file
