# Supplementary material for: A pursuit of lineage-specific and niche-specific proteome features in the world of archaea
Source: BMC Genomics. 2012 Jun 12;13:236. doi: 10.1186/1471-2164-13-236 (PMC3416665; doi:10.1186/1471-2164-13-236)
Supplement: Additional data file1 — Detail information about all archaea under study. [file 1471-2164-13-236-S1.pdf]

Additional data file1: Detail informations about 69 archaea under study

Organism Name	Group	Class	Order	Genome Size (Mb)	GC Content (%)	Temp. range	habitat	oxygen requirement
Aeropyrum pernix K1	CR	TP	desulfurococcales	1.7	56.3	hyperthermophile	***	aerobic
Desulfurococcus kamchatkensis 1221n				1.4	45.3	Hyperthermophilic	Aquatic	Anaerobic
Hyperthermus butylicus DSM 5456				1.7	53.7	Hyperthermophilic	Aquatic	Anaerobic
Staphylothermus marinus F1				1.6	35.7	Hyperthermophilic	Specialized	Anaerobic
Ignicoccus hospitalis KIN4/I				1.3	56.5	Hyperthermophilic, Acidophilic	Aquatic	Anaerobic
Caldivirga maquilingensis IC-167			thermoproteales	2.1	43.1	Hyperthermophilic	Specialized	Micro-aerophilic
Pyrobaculum aerophilum str. IM2				2.2	51.4	Hyperthermophilic, Acidophilic	Aquatic	Facultative
Pyrobaculum arsenaticum DSM 13514				2.1	55.1	Hyperthermophilic	Aquatic	Anaerobic
Pyrobaculum calidifontis JCM 11548				2.0	57.2	Hyperthermophilic	Specialized	Facultative
Pyrobaculum islandicum DSM 4184				1.8	49.6	Thermophilic	Specialized	Anaerobic
Thermofilum pendens Hrk 5				1.8	57.6	Hyperthermophilic	Specialized	Anaerobic
Thermoproteus neutrophilus V24Sta				1.8	59.9	Hyperthermophilic	Specialized	Anaerobic
Metallosphaera sedula DSM 5348			sulfolobales	2.2	46.2	Thermophilic	Specialized	Aerobic
Sulfolobus acidocaldarius DSM 639				2.2	36.7	Thermophilic	Specialized	Aerobic
Sulfolobus islandicus L.S.2.15				2.7	35.1	Hyperthermophilic	Specialized	Aerobic
Sulfolobus islandicus M.14.25				2.6	35.1	Hyperthermophilic	Specialized	Aerobic
Sulfolobus islandicus M.16.27				2.7	35.0	Hyperthermophilic	Specialized	Aerobic
Sulfolobus islandicus M.16.4				2.6	35.0	Hyperthermophilic	Specialized	Aerobic
Sulfolobus islandicus Y.G.57.14				2.7	35.4	Hyperthermophilic	Specialized	Aerobic
Sulfolobus islandicus Y.N.15.51				2.8	35.3	Hyperthermophilic	Specialized	Aerobic
Sulfolobus solfataricus P2				3.0	35.8	Thermophilic	Specialized	Aerobic
Sulfolobus tokodaii str. 7				2.7	32.8	Thermophilic	Specialized	Aerobic
Nitrosopumilus maritimus SCM1	TH			1.6	34.2	Mesophilic	Aquatic	Aerobic
Archaeoglobus fulgidus DSM 4304	EU	AG		2.2	48.6	Hyperthermophilic	Aquatic	Anaerobic
Haloarcula marismortui ATCC 43049		HA		4.3	61.1	Mesophilic	Aquatic	Aerobic
Halobacterium salinarum R1				2.7	65.7	Mesophilic	Specialized	Anaerobic
Halobacterium sp. NRC-1				2.6	65.9	Mesophilic	Specialized	Facultative

Halomicrobium mukohataei DSM 12286				3.3	65.5	Mesophilic	Specialized	Facultative
Haloquadratum walsbyi DSM 16790				3.2	47.9	***	Aquatic	***
Halorhabdus utahensis DSM 12940				3.1	62.9	Mesophilic	Terrestrial	Aerobic
Halorubrum lacusprofundi ATCC 49239				3.7	64.0	Mesophilic	Aquatic	Aerobic
Natronomonas pharaonis DSM 2160				2.8	63.1	***	Aquatic	Aerobic
Methanocaldococcus fervens AG86		MC		1.5		Hyperthermophilic	Specialized	Anaerobic
Methanocaldococcus jannaschii DSM 2661				1.8	31.3	Thermophilic	Aquatic	Anaerobic
Methanococcus aeolicus Nankai-3				1.6	30.0	Mesophilic	Aquatic	Anaerobic
Methanocaldococcus vulcanius M7				1.7	31.6	Hyperthermophilic	Specialized	Anaerobic
Methanococcus maripaludis C5				1.8	33.0	Mesophilic	Aquatic	Anaerobic
Methanococcus maripaludis C6				1.7	33.4	Mesophilic	Aquatic	Anaerobic
Methanococcus maripaludis C7				1.8	33.3	Mesophilic	Aquatic	Anaerobic
Methanococcus maripaludis S2				1.7	33.1	Mesophilic	Aquatic	Anaerobic
Methanococcus vannielii SB				1.7	31.3	Mesophilic	Aquatic	Anaerobic
Methanocella paludicola SANAE				3.0	54.9	Mesophilic	***	***
Candidatus Methanoregula boonei 6A8		MM		2.5	54.5	Mesophilic	Terrestrial	Anaerobic
Methanocorpusculum labreanum Z				1.8	50.0	Mesophilic	Aquatic	Anaerobic
Methanoculleus marisnigri JR1				2.5	62.1	Mesophilic	Aquatic	Anaerobic
Methanococcoides burtonii DSM 6242				2.6	40.8	Mesophilic	Aquatic	Anaerobic
Methanosaeta thermophila PT				1.9	53.5	Thermophilic	***	Anaerobic
Methanosarcina acetivorans C2A				5.8	42.7	Mesophilic	Aquatic	Anaerobic
Methanosarcina barkeri str. Fusaro				4.8	39.2	Mesophilic	Multiple	Anaerobic
Methanosarcina mazei Go1				4.1	41.5	Mesophilic	Multiple	Anaerobic
Methanosphaerula palustris E1-9c				2.9	55.4	Mesophilic	Specialized	Anaerobic
Methanospirillum hungatei JF-1				3.5	45.1	Mesophilic	Multiple	Anaerobic
Methanobrevibacter smithii ATCC 35061		MB		1.9	31.0	Mesophilic	Multiple	Anaerobic
Methanosphaera stadtmanae DSM 3091				1.8	27.6	Mesophilic	Host-associated	Anaerobic
Methanothermobacter thermautotrophicus str. Delta H				1.8	49.5	Thermophilic	Specialized	Anaerobic

Methanopyrus kandleri AV19		MP		1.7	61.2	Hyperthermophilic	Specialized	Anaerobic
Pyrococcus abyssi GE5		TC		1.8	44.7	Thermophilic	Aquatic	Anaerobic
Pyrococcus furiosus DSM 3638				1.9	40.8	Thermophilic	Aquatic	Anaerobic
Pyrococcus horikoshii OT3				1.7	41.9	Thermophilic	Aquatic	Anaerobic
Thermococcus gammatolerans EJ3				2.0	51.3	Hyperthermophilic	Specialized	Anaerobic
Thermococcus kodakarensis KOD1				2.1	52.0	Hyperthermophilic	Specialized	Anaerobic
Thermococcus onnurineus NA1				1.8	51.3	Hyperthermophilic	Terrestrial	Anaerobic
Thermococcus sibiricus MM 739				1.8	***	Hyperthermophilic		Anaerobic
Thermoplasma acidophilum DSM 1728		TL		1.6	46.0	Thermophilic	Specialized	Facultative
Thermoplasma volcanium GSS1				1.6	39.9	Thermophilic	Specialized	Facultative
Picrophilus torridus DSM 9790				1.5	36.0	Thermophilic	Specialized	Aerobic
uncultured methanogenic archaeon RC-I		UN		3.2	54.6	Mesophilic	Host-associated	***
Nanoarchaeum equitans Kin4-M	NA			0.5	31.6	Hyperthermophilic	Host-associated	Anaerobic
Candidatus Korarchaeum cryptofilum OPF8	KO			1.6	49.0	Thermophilic	Specialized	Anaerobic

CR- Crenarchaeota, TH- Thaumarchaeota, EU- Euryarchaeota, NA- Nanoarchaeota, KO- Korarchaeota, TP- Thermoprotei, AG- Archaeoglobus, HA- Halobacteria, MC- Methanococci, MM- Methanomicrobia, MB- Methanobacteria, MP- Methanopyri, TC- Thermococci, TL- Thermoplasmata, UN- Uncultured methanogen
