# Supplementary material for: A pursuit of lineage-specific and niche-specific proteome features in the world of archaea
Source: BMC Genomics. 2012 Jun 12;13:236. doi: 10.1186/1471-2164-13-236 (PMC3416665; doi:10.1186/1471-2164-13-236)
Supplement: Additional data file2 — Details of COGs category. [file 1471-2164-13-236-S2.pdf]

Additional data file2: Details of COG category

Group	Code	Function
Information storage and processing	J	Translation, ribosomal structure and biogenesis
	A	RNA processing and modification
	K	Transcription
	L	Replication, recombination and repair
	B	Chromatin structure and dynamics
Cellular processes and signaling	D	Cell cycle control, cell division, chromosome partitioning
	Y	Nuclear structure
	V	Defense mechanisms
	T	Signal transduction mechanisms
	M	Cell wall/membrane/envelope biogenesis
	N	Cell motility
	Z	Cytoskeleton
	W	Extracellular structures
	U	Intracellular trafficking, secretion, and vesicular transport
	O	Posttranslational modification, protein turnover, chaperones
Metabolism	C	Energy production and conversion
	G	Carbohydrate transport and metabolism
	E	Amino acid transport and metabolism
	F	Nucleotide transport and metabolism
	H	Coenzyme transport and metabolism
	I	Lipid transport and metabolism
	P	Inorganic ion transport and metabolism
	Q	Secondary metabolites biosynthesis, transport and catabolism
Poorly characterized	R	General function prediction only
	S	Function unknown
