# Supplementary material for: A pursuit of lineage-specific and niche-specific proteome features in the world of archaea
Source: BMC Genomics. 2012 Jun 12;13:236. doi: 10.1186/1471-2164-13-236 (PMC3416665; doi:10.1186/1471-2164-13-236)
Supplement: Additional data file3 — Details of all sulphur metabolising and methanogenic archaea under study. [file 1471-2164-13-236-S3.pdf]

Additional data file 3: List of sulphur metabolising and methanogenic archaea with details

Metabolic Adaptation	Phyla	Thermal adaptation	Organism name	Abbr.	Size (Mb)	GC (%)
Sulphur-metabolism	Crenarchaeota	Hyperthermophilic	*Caldivirga maquilingensis IC-167*	C.maq	2.1	43.1
			Hyperthermus butylicus DSM 5456	H.but	1.7	53.7
			Ignicoccus hospitalis KIN4/I	I.hos	1.3	56.5
			Pyrobaculum arsenaticum DSM 13514	P.ars	2.1	55.1
			Staphylothermus marinus F1	S.mar	1.6	35.7
			Sulfolobus islandicus L.S.2.15	S.isl	2.7	35.1
			Sulfolobus islandicus M.14.25	S.isa	2.6	35.1
			Sulfolobus islandicus M.16.27	S.isn	2.7	35.0
			Sulfolobus islandicus M.16.4	S.isd	2.6	35.0
			Sulfolobus islandicus Y.G.57.14	S.isi	2.7	35.4
			Sulfolobus islandicus Y.N.15.51	S.isc	2.8	35.3
			Thermoproteus neutrophilus V24Sta	T.neu	1.8	59.9
			Thermofilum pendens Hrk 5	T.pen	1.8	57.6
		Thermophilic	*Metallosphaera sedula DSM 5348*	M.sed	2.2	46.2
			Pyrobaculum islandicum DSM 4184	P.isl	1.8	49.6
			Sulfolobus acidocaldarius DSM 639	S.aci	2.2	36.7
			Sulfolobus solfataricus P2	S.sol	3.0	35.8
			Sulfolobus tokodaii str. 7	S.tok	2.7	32.8
	Euryarchaeota		*Pyrococcus abyssi GE5*	P.aby	1.8	44.7
			Pyrococcus horikoshii OT3	P.hor	1.7	41.9
			Pyrococcus furiosus DSM 3638	P.fur	1.9	40.8
		Hyper-thermophilic	*Thermococcus gammatolerans EJ3*	T.gam	2.0	51.3
			Thermococcus kodakarensis KOD1	T.kod	2.1	52.0
			Thermococcus onnurineus NA1	T.onn	1.8	51.3
			Thermococcus sibiricus MM 739	T.sib	1.8	40.0
			Archaeoglobus fulgidus DSM 4304	A.ful	2.2	49.0

Metabolic adaptation	Phyla	Thermal adaptation	Organism name	Abbr.	Size (MB)	GC (%)
Methanogen	Euryarchaeota	Mesophilic	*Methanosarcina acetivorans C2A*	M.ace	5.7	42.7
			Methanosarcina mazei Go1	M.maz	4.1	41.5
			Methanococcus maripaludis S2	M.mar	1.7	33.1
			Methanococcus aeolicus Nankai-3	M.aeo	1.6	30.0
			Methanococcus maripaludis C6	M.mac	1.7	33.4
			Methanococcus vannielii SB	M.van	1.7	31.3
			Methanobrevibacter smithii ATCC 35061	M.smi	1.9	31.0
			Methanococcoides burtonii DSM 6242	M.bur	2.6	40.8
			Methanocorpusculum labreanum Z	M.lab	1.8	50.0
			Methanoculleus marisnigri JR1	M.maj	2.5	62.1
			Methanosarcina barkeri str. Fusaro	M.bar	4.8	39.2
			Methanosphaera stadtmanae DSM 3091	M.sta	1.8	27.6
			Methanosphaerula palustris E1-9c	M.pal	2.9	55.4
			Methanospirillum hungatei JF-1	M.hun	3.5	45.1
			Uncultured methanogenic archaeon RC-I	M.arc	3.2	54.6
			Candidatus Methanoregula boonei 6A8	M.boo	2.5	54.5
			Methanococcus maripaludis C5	M.mai	1.8	33.0
			Methanococcus maripaludis C7	M.mal	1.8	33.3
			Methanocella paludicola SANAE	M.pas	3.0	54.9
		Thermophilic	*Methanocaldococcus fervens AG86*	M.fer	1.5	32.0
			Methanocaldococcus jannaschii DSM 2661	M.jan	1.8	31.3
			Methanothermobacter thermautotrophicus str. Delta H	M.del	1.8	49.5
			Methanosaeta thermophila PT	M.the	1.9	53.5
			Methanopyrus kandleri AV19	M.kan	1.7	61.2
			Methanocaldococcus vulcanius M7	M.vul	1.7	31.6
